# Around the Hospitals

**Published:** 1892-07-16

**Authors:** 


### AROUND THE HOSPITALS.

**The Pembrokeshire and Haverfordwest Infirmary** have issued a report that shows that since its establishment no more useful work has been accomplished than that recorded for the past year. Whilst the number of patients was larger, a like increase in expenditure was not incurred. This the Board acknowledge as due to the excellent management of the Matron. The balance on the accounts due to the Treasurer continues to increase, and to diminish this it is proposed that the number of tickets necessary to admit an in-patient should be raised from eight to nine. Even were this done, the value of nine tickets only represents £1 11s. 6d., whereas the actual cost of a patient averages £2. It seems obvious that under such a system of admission the adverse balance must continue to increase. At the annual meeting of the institution, Archdeacon Hilbers stated that the southern part of the county, from which a large number of patients were received, was very inadequately represented by subscribers, the contributions thence being limited to the generous few. Before the next annual meeting we trust he will be able to report differently.

**The Charing Cross Hospital** presents an excellent report for 1891. Increase in the number of patients, legacies, and annual subscriptions is shown. The report itself is admirably drawn up. All salient points are well brought out, and the whole arrangement is clear and concise. Hospital reports admit of considerable difference of treatment, and are frequently either too bald or too diffused as to matter. A large experience in literature of this kind causes us to be very grateful for systematic arrangements of institution records. During the past year the hospital has received £3,419 in legacies. The convalescent home account amounts to £5,890, and Mr. Passmore Edwards, the generous donor of £5,000 of this amount has promised a further subscription for the same purpose. The number of donation boxes held on behalf of this hospital is a special feature, and we learn that applications for others continues to increase, showing that the work of the hospital is much appreciated. The endowment fund of the institution is so small that the large income necessary to maintain it has to be met by voluntary subscription. There is now a deficiency of £9,381 to be met, and the increased generosity of the public alone will enable the council to continue the full amount of useful work possible.

**The Mary Wardell Convalescent Home for Scarlet Fever,** at Stanmore, occupies a unique position, and supplies a decided want. It was established upwards of seven years ago, and during that time over 15,000 patients have been benefited. There are a fair number of Convalescent Homes open to Metropolitan and other patients, but none of these save those under the Metropolitan Asylums Board will receive scarlet fever convalescents, and as change of air is most beneficial towards recovery from this disease, the Home has a very useful mission field. By referring to the list of admissions, we find that patients of most classes have been admitted during the past year, the majority of these being children. The scale of charges are varied to meet the requirements of the case, and one benefactor of the Home takes upon himself the charge of assisting those patients who cannot well meet even the lowest fees The fees do not cover quite one-half of the expenses of the Home. Subscriptions and donations are therefore necessary, and are liberally given. Nevertheless, a small debt still remains to be cleared off the accounts, part of which was incurred in the erection of a new block, consisting of waiting, disinfecting, and bath rooms, and servants' quarters. Miss Wardell is building, at her own cost, a residence for herself adjoining the Home, to become ultimately the property of the institution.

